# The mediating role of intergroup anxiety in the relationship between meta-stereotype and achievement motive among adolescents in rural–urban integration areas: a cross-sectional study

**DOI:** 10.3389/fpsyg.2025.1599706

**Published:** 2025-06-18

**Authors:** Chuanyong Guo, Mingying Li, Limin Hou

**Affiliations:** ^1^Faculty of Education, Guangxi Normal University, Guilin, Guangxi, China; ^2^Nanshan Zhonggong Street Junior High School, Jinan, Shandong, China; ^3^Department of Hematology, Qilu Hospital of Shandong University, Cheeloo College of Medicine, Shandong University, Jinan, Shandong, China

**Keywords:** rural–urban integration, meta-stereotype, intergroup anxiety, achievement motive, education

## Abstract

**Background:**

Adolescence is a crucial period for identity formation and the development of achievement motivation. In the context of rural–urban integration, adolescents encounter diverse social, cultural, and economic dynamics, which complicate their developmental processes through intergroup relations and perceptions. Meta-stereotypes represent a key factor that influences adolescent motivation and behavior. This study aims to examine the mediating role of intergroup anxiety in the relationship between meta-stereotypes and achievement motivation among adolescents in rural–urban integration areas.

**Methods:**

This cross-sectional study included 396 adolescents (aged 12–15 years; mean age = 13.89, SD = 0.98; 48.74% female) from a middle school in the rural–urban integration area. Participants completed self-report measures assessing intergroup anxiety, achievement motivation, and meta-stereotype activation. A mediation analysis with a bias-corrected bootstrapping procedure (5,000 draws) was conducted to assess whether intergroup anxiety mediates the relationship between meta-stereotypes and achievement motivation.

**Results:**

Achievement motivation was negatively correlated with meta-stereotypes (*r* = −0.12, *p* = 0.009), age (*r* = −0.13, *p* = 0.004), years of education (*r* = −0.14, *p* = 0.002), and intergroup anxiety (*r* = −0.30, *p* < 0.001). Intergroup anxiety was positively correlated with meta-stereotypes (*r* = 0.22, *p* < 0.001) and sex (*r* = 0.08, *p* = 0.047). Across all participants, meta-stereotype activation significantly increased intergroup anxiety (*p* < 0.001) and significantly decreased achievement motivation (*p* = 0.019). Rural adolescents exhibited the same pattern, while urban adolescents did not show significant changes. The 95% bootstrap confidence interval for the indirect effect of intergroup anxiety was [−0.201, −0.065], indicating a significant mediation effect. Subgroup analysis revealed no mediating effect among urban adolescents, while rural adolescents demonstrated significant partial mediation by intergroup anxiety on both the motivation to approach success and the motivation to avoid failure.

**Conclusion:**

This study explored the interplay among meta-stereotypes, intergroup anxiety, and achievement motivation in adolescents from rural–urban integration areas in China. The findings offer important insights for educational strategies and psychological interventions aimed at enhancing interpersonal relationships and academic confidence among rural adolescents in these areas.

## 1 Introduction

Adolescence is a critical developmental stage marked by substantial psychological, social, and academic challenges. The ongoing transition from rural to urban life is particularly significant, especially in regions undergoing rapid rural–urban integration. In such contexts, adolescents often experience profound shifts in social norms, educational expectations, and peer group structures. These changes often lead to identity confusion, social disconnection, and motivational instability, making this population especially vulnerable to psychological stressors. Despite its importance, the unique psychological and motivational challenges associated with this transition remain underexplored.

Adolescents living in rural–urban integration areas face distinct contextual pressures compared to their peers in purely rural or urban settings. Prior research has shown that students from rural backgrounds may have limited access to academic resources and support, contributing to lower self-efficacy and reduced achievement motivation (Ma et al., 2021). Conversely, students newly entering urban educational settings may be confronted with social comparison, cultural marginalization, and stereotype-based exclusion, all of which are known to undermine academic confidence and engagement (Ma and Wang, 2025). These environmental dynamics influence how adolescents interpret their social experiences and, in turn, shape their motivational trajectories.

Understanding these challenges, particularly the psychological and motivational processes involved, such as achievement motivation, is essential. “Motivation is the process whereby goal-directed activities are initiated and sustained” (Cook and Artino, 2016). Achievement motive is an important issue of concern to psychologists and individuals in the field of education because it is related to academic self-concept (Marsh and Ayotte, 2003) and academic self-efficacy (Bong and Skaalvik, 2003). According to achievement theory, the scores of the motivation to pursue success (approach motive) subscale of the Achievement Motivation Scale (AMS) are positively correlated with test scores, while the scores of the motivation to avoid failure (avoidance motive) subscale are negatively correlated with the scores (Rand, 1978). Based on these conceptualizations of motivation, a measurement tool was developed to assess the two dimensions (approach motive and avoidance motive) of motivation. Specifically, approach motive refers to behavior that is motivated by positive stimuli (objects, events, possibilities) or moves toward positive stimuli, while avoidance motive refers to behavior that is motivated by negative stimuli (objects, events, possibilities) or moves toward negative stimuli (Elliot, 2006).

School environment has been shown to play an important role in achievement motivation as well as academic achievement of young adolescents (Chetri, 2014; Wang and Eccles, 2013; Yousefy et al., 2012). Achievement motivation results from the continuous interaction among students’ perceptions of the learning environment, learning behaviors, and contextual factors (Pelaccia and Viau, 2017). One such contextual factor is meta-stereotype, defined as an individual’s perception of how their social group is perceived by others. Research has demonstrated that the academic and career motivations of underrepresented and stigmatized students are broadly influenced by meta-stereotypes, for instance, the presence of negative meta-stereotypes is potentially relevant for students’ self-concept and intergroup interactions in the classroom among Turkish-origin students in Germany (Haase et al., 2024). In addition, a growing body of literature has shown that meta-stereotype affects student learning and impairs performance in China through several psychological mechanisms. Specifically, migrant children are more susceptible to meta-stereotypes and engage in frustration and aggressive behaviors than dominant groups (Huang et al., 2019). The deleterious effects of negative meta-stereotype on working memory and intergroup anxiety among migrant children contributed to the declines of working memory at medium level of difficulty (Sun et al., 2015); and positive meta-stereotypes had a choking under-pressure effect on cognitive performance in Chinese migrant children and rural college students, among which negative emotions may be an important mediating factor (He et al., 2024). Although interventions exist to mitigate these effects (Inzlicht and Schmader, 2012; Yeager and Walton, 2011), the underlying mechanisms, particularly in diverse sociocultural settings such as rural–urban integration zones, remain insufficiently understood.

Another critical mechanism is intergroup anxiety, which refers to the discomfort or tension experienced during interactions with members of different social or cultural groups (Anjum et al., 2022; Li and Rose, 2017). Adolescents in transitional environments often navigate between conflicting social identities and may feel excluded or misjudged by out-group peers. Previous studies have suggested that adolescents who are aware of negative meta-stereotypes about their rural background may internalize these views, leading to increased social vigilance and anxiety during intergroup interactions (Sosoo et al., 2020; Xu et al., 2021). Intergroup anxiety, in turn, has been shown to diminish working memory capacity and reduce task persistence, both of which are critical components of achievement motivation (MacInnis and Page-Gould, 2015; Paolini et al., 2018; Schmader et al., 2008). Although research on intergroup anxiety and achievement motivation in rural–urban integration areas remains limited, existing studies suggest that culturally diverse environments can heighten intergroup anxiety among adolescents unfamiliar with such diversity (Baumert et al., 2024; Kim and Kim, 2024). Moreover, the role of intergroup anxiety in shaping academic outcomes within these unique sociocultural contexts remains underexplored, as prior research has largely addressed generalized effects across group types (Brumariu et al., 2023; Rosenthal et al., 2016).

Despite these insights, the mediating role of intergroup anxiety in the relationship between meta-stereotypes and achievement motivation remains underexamined, particularly in rural–urban integration contexts. This study seeks to address this gap by exploring how adolescents’ perceptions of group status (via meta-stereotypes) interact with their intergroup anxiety to influence academic motivation and performance. Clarifying these relationships may offer both theoretical insights and practical guidance for the field of educational psychology. The findings may inform the design of culturally sensitive educational policies and school-based interventions aimed at reducing intergroup anxiety and fostering inclusive learning environments. By promoting a sense of belonging and recognizing individual strengths, schools in rural–urban integration areas can help rural students overcome stereotype-based barriers, improve interpersonal connections, and cultivate stronger academic motivation.

## 2 Materials and methods

### 2.1 Participants and procedure

A total of 396 participants (203 males and 193 females) aged 12–15 years (*M* = 13.89, *SD* = 0.98) were recruited from a middle school in the suburbs of N District, J City, S Province, which has a relatively high level of urbanization. The students of this school come from the surrounding rural areas and J City, and it has the typical characteristics of a middle school in the rural–urban integration areas. The inclusion criteria for the subjects were that the subjects had completed at least 6 years of formal education, provided written informed consent, and obtained the consent of their parents or guardians. The criterion was to ensure that the adolescents included in this study had completed primary school, could fully and independently participate and complete the questionnaire, and that the research group was as homogeneous as possible without interference from other factors. Exclusion criteria included individuals with Chinese character literacy difficulties due to physical or language barriers and individuals whose guardians or themselves refused to participate. Participants were from the first grade (35.86%), second grade (24.75%), and third grade (39.39%). Of all the participants, 78.03% were from rural areas and 21.97% were from urban areas, as rural adolescents constitute the majority of students in schools of rural–urban integration areas (Table 1). After obtaining informed consent, participants were randomly assigned to either the meta-stereotype activation (meta-stereotype) group or the meta-stereotype non-activation (control) group using a computerized randomization procedure to ensure equal assignment probability.

**Table 1:** Descriptive statistics of the study sample.

Variable	Total		Control		Meta-stereotype	
	*n*	%	*n*	%	*n*	%
Total	396	100	199	50.253	197	49.747
Age (Years)						
12	24	6.061	12	50.000	12	50.000
13	141	35.606	68	48.227	73	51.773
14	84	21.212	45	53.571	39	46.429
15	147	37.121	74	50.340	73	49.660
Sex						
Male	203	51.263	102	50.246	101	49.754
Female	193	48.737	97	50.259	96	49.741
Education years						
Grade 1	142	35.859	72	50.704	70	49.296
Grade 2	98	24.747	50	51.020	48	48.980
Grade 3	156	39.394	77	49.359	79	50.641
Residential area						
Rural	309	78.030	163	52.751	146	47.249
Urban	87	21.970	36	41.379	51	58.621

% in Total group vs. 396; % in other group vs. relative total Number (n).

The data were collected during the students’ school hours in the form of paper questionnaires. After the meta-stereotype induction, the participants completed the intergroup anxiety questionnaire and the achievement motivation questionnaire. Finally, participants provided background information, including age and household registration. At the end of the experiment, the experimental design was explained to the participants, and the purpose of the experiment was clarified to mitigate any potential adverse effects. Participants were provided with emotional regulation strategies, and small gifts were given to thank them for their positive co-operation. All participants completed assessments of meta-stereotype negativity (focal predictor), intergroup anxiety (proposed mediator), and achievement motivation (proposed outcome variable).

### 2.2 Demographic information

Demographic information was collected using a self-developed questionnaire. The questionnaire gathered data on sex, age, and years of education. Sex was coded as a dichotomous variable: 1 for male and 2 for female. Age was recorded based on the year of birth. Years of education were measured as a continuous variable, representing the total number of years of schooling starting from the first grade of junior high school.

### 2.3 Meta-stereotype

According to Owuamalam and Zagefka (2013), negative meta-stereotypes are activated when participants provide adjectives based on specific instructions. Since meta-stereotypes are a set of ideas about the characteristics, attributes, and behaviors of a specific group, open-ended questions were used to ask the participants about their negative impressions of rural students. The instructions for the meta-stereotype group were: “What negative opinions do you think urban students have about rural students (e.g., lifestyle, study habits, personality traits)? Please describe them using adjectives.” The instructions for the control group were: “What do you think of the current development of science and technology? Please try to describe them with some adjectives.” The questionnaires were administered collectively by the class teachers, with the assistance of a graduate student in psychology.

### 2.4 Intergroup anxiety

Based on the instrument developed by Stephan and Stephan (1985) to measure intergroup anxiety, the group references were modified to “familiar people” and “strangers” in accordance with the study’s context. The questionnaire consisted of 10 questions using a 10-point scoring system, with items 3, 4, and 5 reverse coded. Participants rated their emotional responses when interacting with strangers versus familiar individuals using the following 10 adjectives: awkward, embarrassed, happy, accepting, confident, irritable, impatient, defensive, suspicious, and cautious. Responses were recorded using a 7-point Likert scale (1 = I would feel like that exactly to 7 = I would not feel like that at all). Total scores were computed by summing the items, with lower scores indicating higher levels of intergroup anxiety. In this study, the scale demonstrated good internal consistency (Cronbach’s *α* = 0.793).

### 2.5 Achievement motives

The achievement motives scale (AMS) (Gjesme and Nygård, 1970; Zhang et al., 2015; Zhou and Dewey, 2024) was used to assess achievement motives. The AMS, grounded in achievement motivation theory, includes items assessing both positive and negative affective responses, as well as items related to situations that supposedly arouse a similar degree of uncertainty as to the possibility of success. The scale contains 30 items describing emotions experienced in achievement-related situations, rated on a 4-point scale (1 = very unsuitable for me; 4 = very suitable for me). The first 15 items are used to calculate the total score for Approach Motive, and the second 15 items are used to calculate the total score for Avoidance Motive. Internal consistency coefficients were 0.871 for the Approach Motive Scale and 0.894 for the Avoidance Motive Scale. The total achievement motivation score was calculated by subtracting the avoidance motive score from the approach motive score. Higher scores indicate stronger tendencies toward either approaching success or avoiding failure. The overall Cronbach’s *α* coefficient for the scale in this study was 0.789.

### 2.6 Data analysis

Statistical analyses were performed using SPSS version 19.0. Descriptive statistics were computed to describe sample characteristics. Pearson correlation analyses were conducted to examine associations among meta-stereotypes, intergroup anxiety, approach motive, avoidance motive, and achievement motive. Based on previous studies (Dong et al., 2023; Laher and Finchilescu, 2010; Sun et al., 2015), we formulated the following *a priori* hypotheses: (1) Participants in the meta-stereotype activation condition would report lower levels of achievement motive than those in the control condition. (2) Participants in the meta-stereotype activation condition would report higher levels of intergroup anxiety than those in the control condition. A *t*-test was used to examine differences between the control group and meta-stereotype group. The significance of direct, indirect, and total effects was evaluated using bootstrapping procedures (bootstrap = 5,000) based on the simple mediation model (MODEL 4). A significance level of *p* < 0.05 was adopted for all analyses.

## 3 Results

### 3.1 Common method bias test

Self-reported data collection may introduce common method bias. Therefore, in addition to using anonymous surveys and reverse-coded items, Harman’s single-factor test was conducted to assess common method bias (Podsakoff et al., 2003). The results indicated that 10 factors with eigenvalues greater than 1 were extracted after rotation. The first factor accounted for 15.414% of the total variance, well below the critical threshold of 40%. Thus, common method bias was not considered a significant concern in this study.

### 3.2 Correlations among study variables

Pearson correlation analyses were conducted among the main study variables (Table 2). Achievement motive scores were negatively correlated with meta-stereotypes (*r* = −0.12, *p* = 0.009), age (*r* = −0.13, *p* = 0.004), years of education (*r* = −0.14, *p* = 0.002), and intergroup anxiety (*r* = −0.30, *p* < 0.001). These findings indicate that lower achievement motivation is associated with meta-stereotype and higher levels of intergroup anxiety. Specifically, intergroup anxiety was negatively correlated with the motivation to approach success (*r* = −0.26, *p* < 0.001) and positively correlated with the motivation to avoid failure (*r* = 0.20, *p* < 0.001), suggesting its detrimental effect on overall achievement motivation (*r* = −0.30, *p* < 0.001). Additionally, as age and educational level increased, adolescents’ achievement motivation declined, particularly in terms of the motivation to approach success (*r* = −0.14 with age, *p* = 0.003; *r* = −0.15 with years of education, *p* = 0.002). Intergroup anxiety was also positively associated with meta-stereotypes (*r* = 0.22, *p* < 0.001) and sex (*r* = 0.08, *p* = 0.047), indicating that both perceived group evaluations and gender differences may contribute to elevated intergroup anxiety.

**Table 2:** Correlations between study variables.

Variable	1	2	3	4	5	6	7	8	9
Meta-stereotype	1								
Age	0.02	1							
Sex	0.00	0.03	1						
Education years	0.01	0.87**	0.08	1					
Residential area	−0.09*	−0.06	−0.08	−0.08*	1				
Intergroup anxiety	0.22***	0.05	0.08*	0.04	0.05	1			
Achievement motive	−0.12**	−0.13**	0.05	−0.14**	−0.04	−0.30***	1		
Approach motive	0.03	−0.14**	0.05	−0.15**	−0.05	−0.26***	0.69**	1	
Avoidance motive	0.19**	0.07	−0.03	0.08	0.01	0.20***	−0.80**	−0.12**	1

*p < 0.05; **p < 0.01; ***p < 0.001.

### 3.3 The influence of meta-stereotypes on intergroup anxiety and achievement motive

An independent-samples t-test was conducted to examine differences in intergroup anxiety and achievement motivation between the meta-stereotype non-activation (control) group and the meta-stereotype activation group (Table 3). Among all participants, activation of meta-stereotype significantly increased intergroup anxiety, *t*(394) = −4.53, *p* < 0.001, *β* = 0.01, and significantly decreased achievement motivation, *t*(394) = 2.36, *p* = 0.019, *β* = 0.35. It also led to a notable increase in the motive to avoid failure, *t*(394) = −3.74, *p* < 0.001, *β* = 0.04. These effects were primarily observed among rural adolescents. In this subgroup, meta-stereotype activation significantly increased intergroup anxiety, *t*(307) = −5.84, *p* < 0.001, *β* < 0.001; decreased achievement motivation, *t*(307) = 2.67, *p* = 0.008, *β* = 0.24; and increased the motive to avoid failure, *t*(307) = −4.61, *p* < 0.001, *β* = 0.004. In contrast, no significant effects were found among urban adolescents.

**Table 3:** Intergroup anxiety and achievement motive.

Variable	Intergroup Anxiety	Achievement Motive	Approach Motive	Avoidance Motive
	*Mean SD* *[95% CI]*	*Mean SD* *[95% CI]*	*Mean SD* *[95% CI]*	*Mean SD* *[95% CI]*
Overall				
Control	44.367 10.539 [42.894, 45.840]	6.397 11.924 [4.730, 8.064]	34.930 7.532 [33.877, 35.983]	28.533 8.561 [27.336, 29.729]
Meta-stereotype	48.853^***^ 9.124 [47.571, 50.135]	3.599^*^ 11.624 [1.966, 5.232]	35.330 6.684 [34.391, 36.269]	31.731^***^ 8.440 [30.545, 32.917]
Rural				
Control	43.828 10.385 [42.222, 45.435]	6.387 11.816 [4.559, 8.214]	34.522 7.602 [33.346, 35.697]	28.135 8.385 [26.838, 29.432]
Meta-stereotype	50.219^***^ 8.633 [48.807, 51.631]	2.966^**^ 10.563 [1.238, 4.694]	35.377 6.511 [34.312, 36.442]	32.411^***^ 7.861 [31.125, 33.697]
Urban				
Control	46.806 11.032 [43.073, 50.538]	6.444 12.573 [2.190, 10.699]	36.778 7.011 [34.406, 39.150]	30.333 9.224 [27.212, 33.454]
Meta-stereotype	44.941 9.441 [42.286, 47.597]	5.412 14.196 [1.419, 9.405]	35.196 7.225 [33.164, 37.228]	29.784 9.735 [27.046, 32.522]

*p < 0.05; **p < 0.01; ***p < 0.001 vs. relative Control group.

### 3.4 Differences in the effects of meta-stereotypes on intergroup anxiety and achievement motive in education years subgroups

We further analyzed rural and urban adolescents across different grade levels and found that participants exhibited varying levels of intergroup anxiety and achievement motive following activation of negative meta-stereotypes (Table 4). Among rural adolescents, activation of meta-stereotypes significantly increased intergroup anxiety in Grade 1 [*t*(110) = −5.81, *p* < 0.001, *β* < 0.001], Grade 2 [*t*(84) = −2.44, *p* = 0.017, *β* = 0.31], and Grade 3 [*t*(109) = −2.09, *p* = 0.039, *β* = 0.46]. In addition, the motive to avoid failure significantly increased in Grade 1 [*t*(110) = −4.11, *p* < 0.001, *β* = 0.017] and Grade 2 [*t*(84) = −2.85, *p* = 0.005, *β* = 0.19] following meta- stereotype activation. Notably, only Grade 1 participants showed a significant decrease in overall achievement motivation after the activation of negative meta-stereotypes [*t*(110) = 3.60, *p* < 0.001, *β* = 0.05], suggesting that younger rural adolescents transitioning into middle school are particularly vulnerable to the effects of meta-stereotypes in rural–urban integration areas. No significant effects were observed among urban adolescents. Moreover, no statistically significant differences were found in the motive to approach success among all participants.

**Table 4:** Intergroup anxiety and achievement motive in education years subgroup.

Variable	Intergroup anxiety		Achievement motive		Approach motive		Avoidance motive	
	Rural	Urban	Rural	Urban	Rural	Urban	Rural	Urban
	*Mean SD* *[95% CI]*	*Mean SD* *[95% CI]*	*Mean SD* *[95% CI]*	*Mean SD* *[95% CI]*	*Mean SD* *[95% CI]*	*Mean SD* *[95% CI]*	*Mean SD* *[95% CI]*	*Mean SD* *[95% CI]*
Grade 1								
Control	43.035 10.257 [40.313, 45.757]	42.133 12.017 [35.479, 48.788]	10.316 11.249 [7.331, 13.301]	9.333 14.430 [1.342, 17.325]	36.351 7.188 [34.444, 38.258]	39.067 7.667 [34.821, 43.312]	26.035 7.136 [24.142, 27.929]	29.733 9.498 [24.474, 34.993]
Meta-stereotype	52.873^***^ 7.371 [50.880, 54.866]	46.867 7.605 [42.655, 51.078]	3.200^***^ 9.542 [0.620, 5.780]	2.333 17.303 [−7.249, 11.915]	35.146 6.857 [33.292, 36.999]	33.867 7.918 [29.482, 38.252]	31.946^***^ 8.068 [29.764, 34.127]	31.533 11.445 [25.196, 37.871]
Grade 2								
Control	41.109 12.356 [37.439, 44.778]	52.500 7.853 [40.004, 64.996]	7.630 9.185 [4.903, 10.358]	7.000 12.329 [−12.618, 26.618]	35.544 7.545 [33.303, 37.784]	38.250 2.872 [33.680, 42.820]	27.913 7.851 [25.582, 30.245]	31.250 9.535 [16.078, 46.422]
Meta-stereotype	46.600^*^ 7.561 [44.182, 49.018]	39.500 7.709 [33.055, 45.945]	3.825 9.850 [0.675, 6.975]	9.500 13.898 [−2.119, 21.119]	36.375 6.356 [34.342, 38.408]	41.375 4.207 [37.858, 44.892]	32.550^**^ 7.118 [30.274, 34.826]	31.875 12.029 [21.829, 41.932]
Grade 3								
Control	46.667 8.077 [44.580, 48.753]	49.588 9.566 [44.670, 54.507]	1.700 12.666 [−1.572, 4.972]	3.765 10.935 [−1.857, 9.387]	32.000 7.465 [30.072, 33.929]	34.412 6.568 [31.035, 37.789]	30.300 9.416 [27.868, 32.732]	30.647 9.460 [25.783, 35.511]
Meta-stereotype	50.196^*^ 9.728 [47.460, 52.932]	45.464 10.419 [41.424, 49.504]	2.039 12.167 [−1.383, 5.461]	5.893 12.547 [1.028, 10.758]	34.843 6.285 [33.076, 36.611]	34.143 6.792 [31.509, 36.776]	32.804 8.307 [30.468, 35.140]	28.250 8.040 [25.133, 31.368]

*p < 0.05; **p < 0.01; ***p < 0.001 vs. relative Control group.

### 3.5 Mediation analysis

Previous results indicated that meta-stereotypes significantly influenced both intergroup anxiety and achievement motive among adolescents in rural–urban integration areas, suggesting a potential mediating role of intergroup anxiety in this relationship. Therefore, a bias-corrected bootstrapping test (with 5,000 draws) based on the mediation model (MODEL 4) was conducted to examine whether intergroup anxiety mediates the relationship between meta-stereotypes and achievement motivation (Table 5). In this analysis, the 95% bootstrap confidence interval for the overall mediation effect of intergroup anxiety on the relationship between meta-stereotypes and achievement motivation among all participants was [−0.201, −0.065], which does not include zero, indicating statistical significance. Additionally, the indirect effects of intergroup anxiety on motivation to approach success and motivation to avoid failure yielded 95% bootstrap confidence intervals of [−0.191, −0.065] and [0.022, 0.137], respectively. These results suggest that intergroup anxiety significantly mediated the effects of meta-stereotypes on both achievement motivation and its subcomponents.

**Table 5:** Multi-group effect decomposition for the mediation model.

Group	Path	Standardized effect	*SE*	*t*	*P*	*95% LLCI*	*95%* *ULCI*
Total	X → M → Y	−0.127*	0.035	—	—	−0.201	−0.065
	X → Y	−0.109	1.167	−1.107	0.269	−3.583	1.002
	X → M → Y1	−0.122*	0.032	—	—	−0.191	−0.065
	X → Y1	0.179	0.708	1.798	0.073	−0.119	2.665
	X → M → Y2	0.074*	0.030	—	—	0.022	0.137
	X → Y2	0.297*	0.866	2.962	0.003	0.862	4.265
Rural	X → M → Y	−0.181*	0.047	—	—	−0.279	−0.097
	X → Y	−0.120	1.300	−1.048	0.296	−3.921	1.197
	X → M → Y1	−0.192*	0.047	—	—	−0.294	−0.109
	X → Y1	0.313*	0.819	2.713	0.007	0.610	3.833
	X → M → Y2	0.082*	0.044	—	—	−0.001	−0.174
	X → Y2	0.426*	0.928	4.609	0.000	2.450	6.102
Urban	X → M → Y	0.052	0.076	—	—	−0.063	0.238
	X → Y	−0.129	2.859	−0.607	0.546	−7.421	3.952
	X → M → Y1	0.049	0.077	—	—	−0.052	0.253
	X → Y1	−0.271	1.513	−1.278	0.205	−4.940	1.075
	X → M → Y2	−0.037	0.056	—	—	−0.171	0.054
	X → Y2	−0.021	2.053	−0.096	0.924	−4.280	3.884

X, meta-stereotype; M, intergroup anxiety; Y, achievement motive; Y1, approach motive; Y2, avoidance motive; *, The mediating effect is significant.

**Figure 1:**
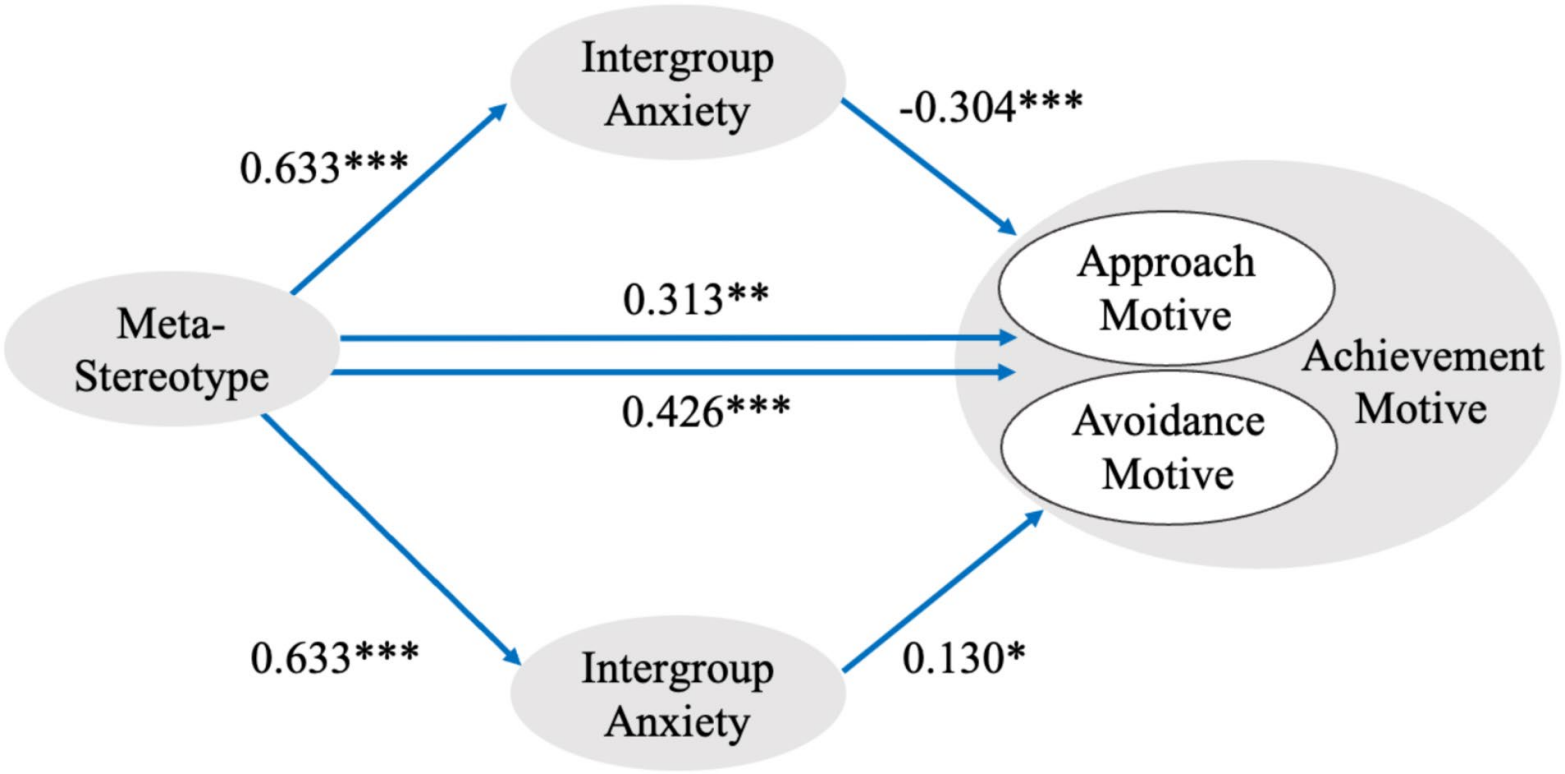
Mediation model path visualization for rural adolescents. *p < 0.05; **p < 0.01; ***p < 0.001.

Similarly, subgroup analyses revealed that intergroup anxiety did not significantly mediate the relationship among urban adolescents. In contrast, rural adolescents exhibited significant partial mediation effects of intergroup anxiety on both sub-dimensions of achievement motive. Specifically, for rural adolescents, the 95% bootstrap confidence interval for the mediating effect of intergroup anxiety on the relationship between meta-stereotypes and achievement motivation was [−0.279, −0.097], not including zero, indicating a significant mediation effect. For the motivation sub-dimensions of avoiding failure and approaching success, the 95% bootstrap confidence intervals of the mediation effect were [−0.294, −0.109] and [−0.174, −0.001], respectively, both intervals excluded zero, further confirming the significance of the mediation effect. Additionally, meta-stereotypes directly influenced both the motive to avoid failure and the motive to approach success. The mediation model is illustrated in Figure 1, depicting the mediating role of intergroup anxiety in the relationship between meta-stereotypes and achievement motivation among rural adolescents.

## 4 Discussion

China’s rapid urbanization has resulted in increasing integration between rural and urban populations. This sociocultural context may unintentionally foster conditions conducive to meta-stereotyping. Rural students are often stereotyped as less educated or less capable. Even within integrated classrooms, implicit biases against rural students may persist. Studies have shown that rural students tend to perform worse academically than their urban peers, even when attending the same schools (Xu et al., 2024). Evidence suggests that simply making students’ rural versus urban identities salient can influence academic performance. The study conducted an experiment with primary school children in China, varying the salience of the student’s hukou (household registration system in China) status during a task. They found that when rural students became aware of their rural identity in a competitive setting, their task performance was negatively affected, which is consistent with meta-stereotype (Afridi et al., 2015). This suggests that the “rural student” identity carries a meta-stereotype (of lower ability or status) that, when emphasized, can undermine confidence and outcomes. In essence, a rural student in an integrated classroom may underperform if they worry that “people like me” aren’t expected to do well. This psychological burden constitutes a significant yet often overlooked barrier to educational integration. Consistent with these findings, our study also revealed that activation of meta-stereotypes, rural adolescents had weaker achievement motivation, especially their motivation to avoid failure became stronger.

Meta-stereotype is not limited to the rural–urban divide in China. Similar patterns have been observed among ethnic minority and low-income students in China. For instance, a study on economically disadvantaged, first-generation college students (typically from rural areas) showed that they also experienced meta-stereotype, referring to the perception that one’s group is viewed as “less capable.” The study found that when these students perceived stereotype threat, intergroup relations on campus deteriorated. Importantly, this effect was mediated by intergroup anxiety. Students who felt stereotyped by peers became anxious in intergroup interactions, which, in turn, resulted in reduced engagement and increased distrust (Jiatong et al., 2022). These findings mirror those observed in junior high school setting: meta-stereotype makes students withdraw or behave defensively, straining the classroom climate. Similarly, our study found that meta-stereotype activation increased intergroup anxiety, particularly among rural adolescents.

A seminal study on migrant children in China directly investigated this mediating effect. The researchers introduced the concept of meta-stereotype, where children are aware that others may hold stereotypes about their group, which is true of rural migrant children in urban schools. Furthermore, when migrant elementary school students were exposed to meta-stereotype activation, their working memory scores decreased significantly (Sun et al., 2015). From a cognitive perspective, anxiety may consume attentional resources and reduce the availability of working memory for goal-directed learning (Schmader et al., 2008). In intergroup contexts, this manifests as lower confidence, reduced persistence, and avoidance of performance situations—mechanisms that plausibly mediate the observed effects of meta-stereotypes on motivation. Given the importance of working memory for learning and test-taking, this impairment is particularly concerning. Most importantly, through structural equation modeling, the researchers found that intergroup anxiety was the mediating mechanism: meta-stereotype increased intergroup anxiety, which, in turn, impaired working memory performance. This finding is important because it empirically verifies the theoretical prediction that intergroup anxiety is a key channel through which meta-stereotype exerts its effects. This finding also highlights that even at a very young age, the psychology of migrant children (rural children) can be affected by group stereotypes (Sun et al., 2015). In line with this, our study confirmed the mediating role of intergroup anxiety in the relationship between meta-stereotype and achievement motive. Also, we found that rural first-grade participants showed a decrease in achievement motivation after activating negative meta-stereotypes, which suggests that meta-stereotypes exert a stronger impact on rural adolescents transitioning into junior high school in rural–urban integrated areas.

Understanding the mediating role of intergroup anxiety offers valuable insights for intervention. If we can reduce the anxiety and improve intergroup relations, we may weaken the effect of meta-stereotype. One approach, demonstrated by Yang et al. (2023), involves strengthening students’ personal identities and self-worth so that meta-stereotypes are less impactful. Yang’s intervention effectively cut the link between group identity and performance by assuring students that their value is not defined by stereotypes. Another effective strategy is to foster positive intergroup contact. According to Allport’s contact theory, positive intergroup interactions reduce prejudice and anxiety (Allport, 1954). In a series of experiments in China, Long et al. (2022) found that imagined contact is important for influencing younger adults’ intergroup anxiety and stereotyping factors that are critical to improving younger adults’ ability to take other group’s perspectives. Although this research focused on college students, the underlying principles can be adapted to secondary school settings: structured cooperative activities where rural and urban students work toward common goals could alleviate anxieties and build mutual respect. Indeed, cross-group friendships in Chinese schools have been shown to enhance attitudes and emotional well-being, which likely makes stereotype-based worries less acute.

In summary, addressing meta-stereotype in education requires confronting both the stereotype and the psychological threat it poses. In practice, this means debiasing the environment (reducing negative stereotypes and differential treatment) and supporting students to manage stress. Schools in rural–urban integration areas should strive to create an atmosphere of inclusion where all students feel they belong and are valued for their individual strengths. In doing so, they can help dismantle the psychological constraints imposed by meta-stereotypes. Interventions that foster empathy and friendships across group lines can lower intergroup anxiety, creating a positive feedback loop of improved relations and academic confidence. As China continues to bridge rural–urban divides, ensuring that meta-stereotypes do not hinder the educational advancement of rural adolescents is both a matter of equity and of tapping the full potential of every adolescent. A decade of research provides a robust foundation for action: it is now up to educators and policymakers to apply these insights so that no adolescent is held back by the shadow of meta-stereotypes.

Although this study provides insights into the interplay between meta-stereotypes, intergroup anxiety, and achievement motive among adolescents in China’s rural–urban integration areas, it still has some limitations. First, the sample was from only one middle school and may not be representative of the wider adolescent population. Second, although the self-report measure was validated, adolescents may have concealed information and its accuracy remains to be verified. In addition, the cross-sectional study design limits our ability to infer causal relationships between the variables studied, thereby limiting the directionality of understanding these relationships. More open quantitative research in psychology and behavioral studies (Yang et al., 2024), coupled with adequate privacy protection measures (Zhang et al., 2025), is essential to ensure the scientific validity and scalability of research findings. Future research should expand the sample to include more adolescent groups in schools in rural–urban integration areas to enhance the generalizability of the findings. Intervention studies aimed at avoiding meta-stereotypes and reducing intergroup anxiety may provide practical insights for improving adolescent achievement motivation. In addition, it is also important to explore these relationships in different cultural contexts. Using mixed methods, combining self-reports with objective assessments, can mitigate the bias inherent in self-report measures and provide a more nuanced perspective on these complex phenomena.

## Data Availability

The original contributions presented in the study are included in the article/supplementary material, further inquiries can be directed to the corresponding author/s.
